# The Role of Hollow Glass Microspheres as Functional Fillers in Fiber-Reinforced Polymer Composites: A Review

**DOI:** 10.3390/ma18214974

**Published:** 2025-10-31

**Authors:** Dehenenet Flatie Tassaw, Marcin Barburski, Bantamlak Birlie Kassie

**Affiliations:** 1 Institute of Architecture of Textiles, Faculty of Textiles and Design, Lodz University of Technology, 116 Zeromskiego Str., 90-924 Lodz, Poland; marcin.barburski@p.lodz.pl; 2 Textile Faculty, Ethiopian Institute of Textile and Fashion Technology, Bahir Dar University, Bahir Dar P.O. Box 1037, Ethiopia; bantamlakbirlie@gmail.com

**Keywords:** hollow glass microsphere, fiber reinforced polymer composite, functional filler, lightweight composites

## Abstract

Fiber-reinforced polymer composites (FRPCs) have gained increasing attention as lightweight structural materials with tailored mechanical, thermal, and functional properties for diverse engineering applications. However, achieving optimal performance requires overcoming challenges such as poor interfacial bonding, high density of conventional fillers, and limitations in multifunctionality. Hollow Glass Microspheres (HGMs), owing to their unique spherical morphology, low density, high strength-to-weight ratio, and tunable physical–chemical characteristics, have emerged as promising functional fillers for FRPCs. This review provides a comprehensive overview of the structural features, chemical composition, and synthesis techniques of HGMs, followed by an outline of FRPCs systems with emphasis on matrix and fiber types, their functional requirements, and the critical role of fillers. The discussion highlights how HGMs influence the mechanical (tensile, flexural and compression strength) properties, thermal (conductivity and insulation) properties, acoustic (sound absorption and transmission) properties, and dielectric performance of FRPCs, enabling weight reduction, improved insulation, and multifunctional capabilities. Reported studies demonstrate that when properly dispersed with an optimal amount, HGMs significantly enhance mechanical properties, thermal stability, and acoustic damping, while maintaining processability. Despite these advantages, challenges remain regarding interfacial adhesion (agglomeration) and filler dispersion. The review concludes by emphasizing the need for advanced surface modification strategies, hybrid filler systems, and sustainable processing methods to fully exploit HGMs in next-generation high-performance FRPCs.

## 1. Introduction

In recent years, fiber-reinforced polymer composites (FRPCs) have been widely employed across various industries, owing to their exceptional properties, including high stiffness, excellent strength-to-weight ratio, and superior resistance to wear and corrosion [1]. The global FRPCs market size was valued at USD 91.21 billion in 2023 and is projected to grow from USD 98.12 billion in 2024 to USD 189.80 billion by 2032 at a CAGR of 7.6% during 2024–2032 [2].

The reinforcement and matrix are the two essential phases of developing a polymer composite [3,4]. The matrix is a component that is incorporated in the reinforcing fiber phase and binds the fibers together. It is used for transferring load from external sources to the reinforcing fibers and the composite through the interface [5], and fillers are added into the compositions, and they are usually the inactive materials which are used in composite materials to decrease material costs, to improve mechanical properties and to improve process ability [6]. In some cases, they serve to impart or improve the following properties: reducing plasticizer absorption [7], changing in dielectric properties [8,9], increasing rigidity and hardness [10,11], reducing noise transmission [12,13], reducing toxicity of combustion products [14,15].

**Figure 1:**
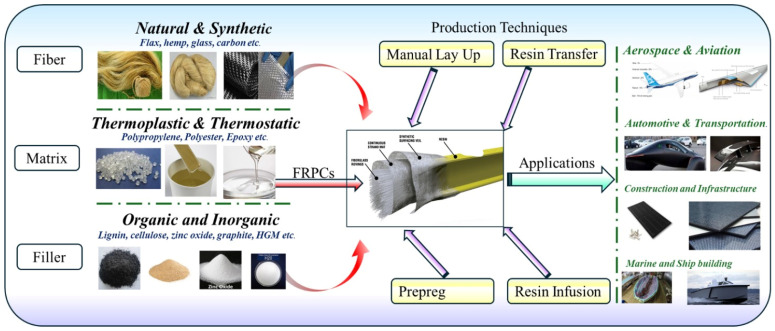
General overview of FRPCs with fillers, its manufacturing techniques and application areas.

The impact of fillers on the composite depends on the filler shape, size, surface area, aspect ratio and distribution of particles in the matrix phase [16]. FRPCs have been integrated into daily life for nearly a century, yet they remain an area of active research and development. Figure 1 below shows the FRPC components, with the production techniques and possible application areas. Ongoing efforts focus on enhancing the performance and functionality of both reinforcement fibers and polymer matrices. Key research directions include improving mechanical properties, promoting environmental sustainability, and increasing economic viability through material innovation and optimized composite design.

Fillers can be classified using various criteria based on their physical state, origin, structure, and functionality. Based on their state of aggregation, fillers are categorized as gaseous, liquid, or solid. In terms of chemical nature, they are broadly classified into organic and inorganic types [4]. According to their functional role in the composite matrix, fillers can be described as reinforcing, strengthening, neutral, or structural [17]. From the perspective of particle size, shape, and morphology, fillers are commonly grouped into four major categories: Dispersed fillers (e.g., powders), fibrous fillers (e.g., fibers, threads, bundles), sheet-like fillers (e.g., films, fabrics, papers, tapes, nets), and volumetric or structural fillers, which possess a continuous three-dimensional framework (e.g., bulk fabrics, felts, skeletal and porous structures) [18,19].

Inorganic fillers have gained more attention in polymer composites in terms of mechanical properties, wear performance, thermal stability, fiber matrix bonding as compared to natural fillers [20]. Such fillers are listed as silicon carbide (SiC), aluminum oxide (Al_2_O_3_), zinc oxide (ZnO), graphite, zeolite, calcium carbonate (CaCO_3_), magnesium hydroxide (Mg(OH)_2_), boron carbide (B_4_C), carbon powder, silicon dioxide (SiO_2_), titanium dioxide (TiO_2_), zirconium dioxide (ZrO_2_) and tungsten carbide (WC) [10].

This review begins with a concise introduction to the fibers and matrices used in FRPCs, outlining their required functionalities and associated challenges, while highlighting the role of fillers in enhancing FRPC performance. The core focus is on summarizing the functional role of HGMs in FRPCs, covering key properties such as mechanical, thermal, acoustic, and dielectric performance. Furthermore, the review provides insights into current research gaps and emerging directions, emphasizing the need for surface modification, hybrid filler systems, and green manufacturing approaches. Future perspectives also include exploring sustainable and bio-based polymer matrices integrated with HGMs to develop lightweight, multifunctional, and eco-friendly composites for advanced engineering applications. This review will be particularly useful for researchers, material scientists, and engineers working in the fields of composite design, functional fillers, and lightweight structural materials. The summarized insights into the role of HGMs as functional fillers can support the development of next-generation FRPCs with improved multifunctional performance.

## 2. Hollow Glass Microspheres (HGMs)

By the late 1930s, solid glass beads made from waste soda–lime glass were incorporated into reflective road paints and later adapted for military uses such as retro-reflective sheets. Beck observed that prolonged exposure to moisture produced microbubbles near the bead surfaces, inspiring the concept of HGMs. In 1963, he patented a two-stage manufacturing process involving frit formulation, milling, and reheating to create single-wall HGMs [21]. Glass microspheres are spherical particles, typically 1–200 μm in diameter, and can be categorized as solid, hollow, or porous. They have been in use for over a century [22,23]. HGMs, also referred to as micro-balloons or glass bubbles, are high-strength, low-density additives composed of water-resistant and chemically stable soda-lime-borosilicate glass. They typically have diameters ranging from 10 to 300 μm and densities between 0.20 and 0.60 g/cm^3^ [24,25,26].

### 2.1. Structure and Composition

**Figure 2:**
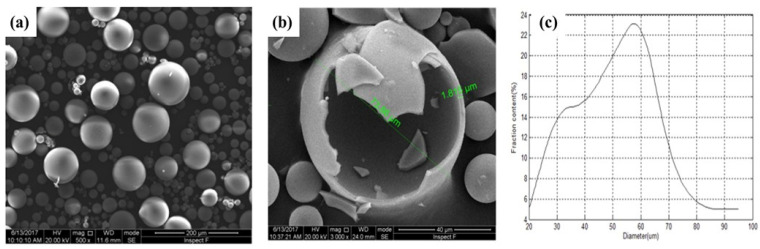
The SEM image of (**a**) HGM, (**b**) cracked HGMs, (**c**) diameter distribution of HGM [30].

HGMs are commonly fabricated from either borosilicate or soda–lime glass. In the borosilicate system, an appropriate composition induces phase separation into two distinct phases: one rich in silica and the other rich in sodium borate [27,28,29]. The wall of HGM is mainly composed of about 80% of SiO_2_ and 20% CaO [30]. Structurally, the wall thickness of hollow spheres determines their crushing strength; as expected, an increase in sphere density corresponds to higher crushing strength. Lightweight hollow glass spheres are chemically stable, non-combustible, non-porous, and exhibit excellent water resistance, making them promising inorganic fillers for fiber-reinforced composites [31]. Figure 2 presents scanning electron microscopy (SEM) images that provide detailed visualization of HGMs and their cavities. The microspheres are perfectly spherical, although their diameters vary within a specific range, and the cross-section of a typical cracked microsphere reveals its distinct hollow structure and uniform wall thickness while the diameter distribution of microspheres are different ranges and levels [30].

### 2.2. Physical and Chemical Properties

The hollow structure and fine spherical shape make HGM have some distinctive properties, such as high compressive strength, low density, low water absorption, low heat conduction, and high chemical resistance [22,32,33,34]. The compressive strength of the HGMs could be changed by controlling the radius and wall thickness of the microspheres [35]. HGMs occupy a relatively low surface area to volume ratio and therefore cause less viscosity build while providing isotropic filling of resin during composite preparation, exhibit low oil absorption, and disperse uniformly within mixtures [36,37]. HGMs typically exhibit a glass transition (softening) (Tg) temperature of around 600 °C; at a temperature above Tg, the material is thermodynamically a liquid that is metastable and of high viscosity. The thermal conductivity of HGMs is also a function of their hollow volume and wall thickness. Their low coefficient of thermal expansion helps to prevent shrinkage during their production and improves the fit and finish as well as reducing noise, vibration and harness for composite material [30,38]. HGM-filled materials are typically characterized by low dielectric constants and low loss tangents due to the void volume of HGMs [39,40].

### 2.3. Synthesis and Manufacturing Techniques

Many different methods are applied for the fabrication of glass microspheres, including the flame synthesis process [41], the liquid droplet method, the dried gel process and the electrical arc plasma [42]. HGMs can be produced by incorporating a blowing agent, such as sodium sulfate or urea, into the glass powder precursor. The choice of blowing agent depends on its decomposition temperature, which should be close to the melting temperature of the selected glass [25,43]. HGMs were synthesized using the flame synthesis method, in which glass particles are fed into an oxy-acetylene or oxygen–methane flame zone. The intense heat softens the glass, enabling it to form spherical shapes. Various blowing agents, most commonly sodium sulfate [44], PVA [45] and urea [42,46] are employed to facilitate hollow structure formation. A blowing agent was applied which, upon decomposition at elevated temperatures, released gas nuclei or bubbles. These bubbles expanded within the softened glass, resulting in the formation of HGMs. A common refining agent is sodium sulfate (Na_2_SO_4_), which melts at 1157 K and decomposes in viscous glass above 1673 K, according to the reaction below [47,48].

$$Na_{2}SO_{4}\to \;\Delta \;Na_{2}O+SO_{3}$$

$$Na_{2}SO_{4}--\to Na_{2}O+SO_{2}+0.5O_{2}$$

**Figure 3:**
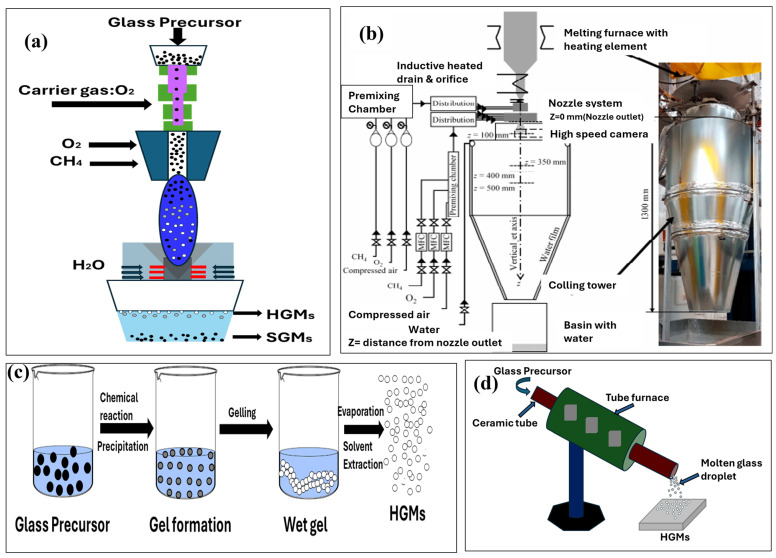
Synthesis techniques of (**a**,**b**) flame/burning synthesis [44,45], (**c**) sol–gel. Created based on information from [49,52], (**d**) tube furnace methods (drawn by authors).

The decomposition behavior of sodium sulfate is strongly influenced by the oxidation state of the glass melt, as the reaction occurs predominantly under oxidizing or mildly reducing conditions at elevated temperatures. For effective production of hollow microspheres, utilizing a low-melting, oxidizing glass composition is advantageous, as it promotes the decomposition of Na_2_SO_4_ within the flame temperature range following atomization [47,48]. The burning process of glass precursor can be performed using Vertical Thermal Flame Process (VTF) [44] as shown in Figure 3a and or a self-designed setup burning tube [44] in Figure 3b. HGMs can also be produced from sol–gel derived-sodium–borosilicate glass specifically formulated for photo-enhanced hydrogen diffusion in hydrogen storage applications, as shown in Figure 3c. In this method, the heat-treated xerogel is suspended and doped with iron sulfate or iron chloride, which serve as both transitional metal dopants and blowing agents. The resulting suspension is spray-dried into granules, which are subsequently flame-sprayed in an oxy–propane flame to form glass microspheres [49]. Tube furnace methods are also used to synthesize HGMs. This was achieved by a tube furnace hot zone, as shown Figure 3d, which relies on heating argon gas in a quartz tube. The setup is capable of simultaneously producing hundreds of high-quality microsphere lasers, without any need to prefilter the particle size, by employing a novel glass injection system. After fabrication, the microspheres are optically pumped via a tapered optical fiber [50]. But other methods like liquid droplet, rotating electrical arc, and argon plasma jet have also been used for the synthesis of HGM [22,51].

## 3. Overview of Fiber-Reinforced Polymer Composites (FRPCs)

FRPCs represent a highly versatile and indispensable class of materials with extensive applications across a wide range of industries. Their adaptability and diverse classification criteria enable the design of tailored solutions to meet specific performance requirements in fields such as civil engineering, aerospace, automotive, and marine sectors. The idea of composites is that of a multi-phase material, a matrix and a reinforcement, bound together so as to have a complete physical interaction, which is defined as “interface”. The characteristics and performance of FRPCs are primarily determined by factors such as the type of reinforcing fiber, the nature of the polymer matrix, the manufacturing process, fiber orientation, and the intended end-use application [53,54,55]. FRPCs are made up of short and continuous natural or synthetic fibers that are organized in both unidirectional and bidirectional configurations with is thermoplastic or thermoset polymer matrix [56,57].

### 3.1. Matrix and Fiber Types

Fiber is a key component of fiber-reinforced composites, embedded within the matrix phase while maintaining its intrinsic properties [58]. Natural, synthetic and hybrid fibers can be used in reinforcement in FRPCs [59,60,61,62]. Together with the matrix, it contributes to the overall performance of the composite. The incorporation of natural fibers into polymer matrices seeks to improve environmental sustainability while mitigating issues related to residue accumulation. Natural fibers, derived from both animal and plant sources, are abundant, non-toxic, renewable, and cost-effective. They also exhibit strong bonding with polymer matrices, resulting in enhanced material properties such as increased ductility, toughness, flexural strength, and impact resistance [63,64,65]. The use of synthetic fibers as reinforcing elements has grown rapidly due to their ability to produce lightweight materials with enhanced strength, modulus, and stiffness [66]. Factors such as fiber’s shape, size, orientation, surface condition, and chemical composition significantly influence the mechanical properties of the composite, including strength, toughness, stiffness, and corrosion resistance [67,68,69].

**Figure 4:**
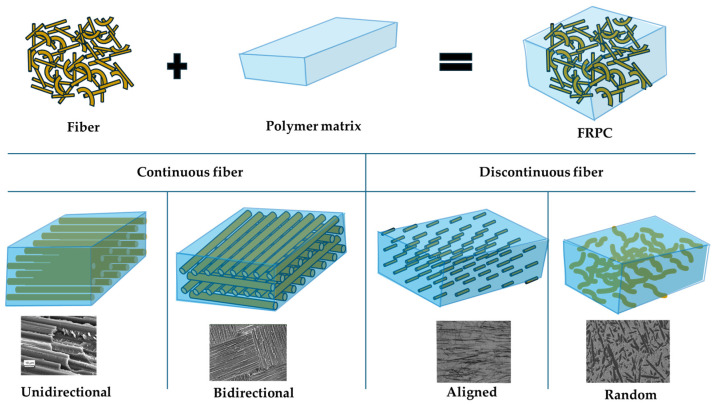
Structure and orientations of fiber in FRPCs.

Polymer matrixes are available in various physical forms such as granules, pellets, sheets, or liquids, depending on the type of polymer. Thermoplastics like polypropylene (PP) and low-density polyethylene (LDPE) are typically supplied as granules and sheets, while the biodegradable thermoplastic polylactic acid (PLA) is available as a liquid, in thin sheet and fiber forms. Among thermosetting polymers, polyester is commonly found as fibers and liquids, whereas epoxy is exclusively supplied as a liquid [66,70]. Composites consist of fibers in the matrix structure and can be classified according to fiber length and orientation, as shown in Figure 4.

Composites with long fiber reinforcements are termed as continuous fiber reinforcement composites, while composites with short fiber reinforcements are termed as discontinuous fiber reinforcement composites. Hybrid fiber-reinforced composites are those where two or more types of fibers are reinforced in a single matrix structure [32]. Fibers can be placed unidirectionally or bidirectionally in the matrix structure of continuous fiber composites, and they take loads from the matrix to the fiber in a very easy and effective way. Discontinuous fibers must have sufficient length for effective load transfer and to restrain the growth of cracks to avoid material failure in the case of brittle matrices [17,71].

### 3.2. Functional Requirements and Challenges

FRPCs are engineered to meet specific functional requirements, such as a high strength-to-weight ratio [72], corrosion resistance [73], durability [74], and design flexibility [75]. These materials are widely used in aerospace, automotive, construction, and sports industries due to their superior mechanical properties and lightweight nature [76,77]. However, several challenges hinder their widespread adoption. These include high production costs [78], difficulty in recycling [79], issues related to fiber–matrix interfacial bonding [80], and long-term performance under environmental stressors such as moisture [81,82], temperature fluctuations [83,84], and UV exposure [85,86]. Furthermore, manufacturing complexity and quality control in large-scale applications remain critical concerns [87]. Addressing these challenges is essential for advancing the performance and sustainability of FRP composites in future applications.

### 3.3. Role of Fillers in FRPCs

**Figure 5:**
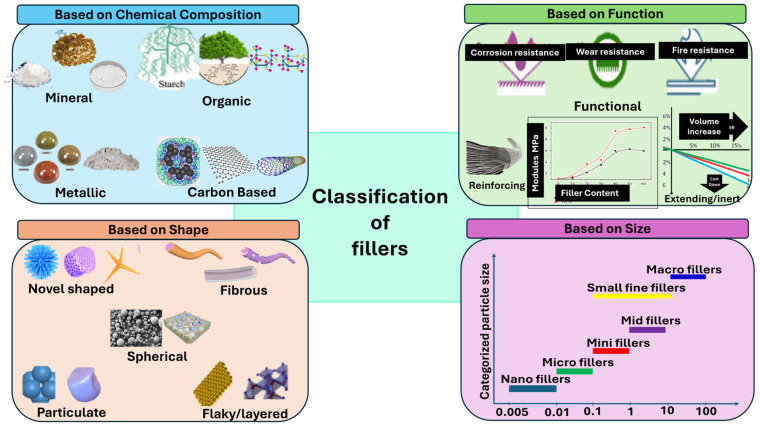
Classification of fillers for composites based on different perspectives.

In modern manufacturing of FRPCs, both organic and inorganic fillers are incorporated not only to enhance structural integrity, improve fracture toughness, extend material service life, reduce weight, and lower production costs, but also to enhance mechanical properties and processability. Fillers can be divided into different categories based on their sources, shapes, size and functionality [88] and the details of the classification are shown in Figure 5. These fillers enable performance characteristics that cannot be achieved by reinforcement and resin components alone [89,90,91].

Several studies have reported that the incorporation of inorganic fillers significantly enhances the mechanical, thermal, and dielectric properties of fiber-reinforced polymer composites. For example, the addition of silica nanoparticles improved the thermal stability, tensile strength, and dielectric performance of polyimide and hybrid epoxy composites at optimal loadings of 2–3 wt%, mainly due to better nanoparticle dispersion using silane coupling agents [92,93]. Likewise, calcium carbonate (CaCO_3_) fillers increased tensile and flexural strength by about 15% and 10%, respectively, in pine fiber–reinforced polyester composites [94]. Incorporating aluminum oxide (Al_2_O_3_) microparticles enhanced the tensile and impact strength of pineapple fiber composites by 23% and 21%, respectively [95], while zinc oxide (ZnO) nanoparticles improved the tensile strength and thermal stability of glass fiber–reinforced epoxy composites by around 18% and 20% [96]. Similarly, HGMs act as functional fillers that not only improve the mechanical and thermal properties of composites but also offer significant weight reduction compared to conventional fillers.

## 4. Functional Role of HGMs in FRPCs

The use of HGMs in composites is creating new opportunities in the composites industry. HGMs consist of a stiff glass hollow sphere filled with inert gas, resulting in some specific properties like low weight, reduced dielectric constant, and reduced thermal conductivity. Solid fillers have a density higher than that of the host resin and add a significant amount of weight to the final polymer composite. This is especially true for highly filled situations such as flame retardant and thermally conductive applications. For example, aluminum trihydrate is used as a flame retardant, and the loading can exceed 70 wt%. The advantage of HGMs changed the paradigm that fillers cause the weight of the polymer composite to increase. HGMs are excellent strength/weight optimizers when they are used in filled polymer systems such as glass fiber (GF), talc, and calcium carbonate-filled thermoplastics. Reducing and replacing a certain percentage of these high-density fillers with HGMs results in weight reduction while significantly maintaining the original mechanical properties of the composite. For instance, thermoplastic olefin (TPO)-based compositions containing large amounts of talc have been successfully modified with HGMs, reducing the density up to 13% while maintaining an acceptable balance of performance and processing characteristics for injection molded automotive parts. HGMs have also been shown to be successfully incorporated into high-temperature polymers, such as polyetherimide, for 10% weight reduction and greater savings in aerospace applications. In comparison with GF-reinforced grades, significant weight savings were achieved with price advantages [33,97].

When incorporating HGMs into a resin system, it is also crucial to accurately determine the volume percentage of all components. Failure to do so may result in excessive removal of the binder (resin) or an unintended dilution of other reinforcing and functional fillers. Another important consideration is the separation behavior; unlike most fillers and pigments that settle at the bottom, HGMs tend to float to the surface of low-viscosity liquids due to their low density. Additionally, HGMs increase cooling rates of polymer resin from the melt through their effect on thermal diffusivity than other inorganic fillers. Increased HGMs weight fraction decreases composite density and composite specific heat capacity, which in turn increases thermal diffusivity and hence cooling rates. Long cooling times incur additional manufacturing costs and can limit production capacity [33].

The incorporation of HGMs into polymer matrices provides an effective route to reduce composite density while maintaining structural performance; however, the full benefit of this lightweight filler is only achieved when interfacial adhesion and filler dispersion are carefully managed. For example, Ajayi et al. reported that modifying HGMs with polypropylene-grafted maleic anhydride (PP-g-MA) significantly enhanced the tensile strength of epoxy foam composites from a 16.35% increase in unmodified systems to 45.31% when using surface-modified HGMs due to improved filler matrix bonding and reduced agglomeration [98]. In addition, several studies have shown that processing parameters such as mixing viscosity, degassing, and curing temperature strongly affect dispersion quality, void content, and consequently the durability of HGM composites. Afolabi et al. demonstrated that applying a controlled degassing step during resin casting of epoxy syntactic foams reduced void content below 5%, improving both tensile and dynamic mechanical properties [99]. Similarly, Berata, Wet al. reported that variation in curing temperature significantly influenced compressive strength, identifying an optimal curing regime around 90 °C [100]. Moreover, inadequate mixing and increased HGM volume fraction led to higher void formation and reduced uniformity within the matrix, consequently decreasing mechanical performance [101]. Collectively, these studies confirm that beyond filler-volume fraction, the optimization of surface modification and processing conditions is critical to minimize agglomeration and enhance the mechanical reliability of HGM-reinforced composites.

**Figure 6:**
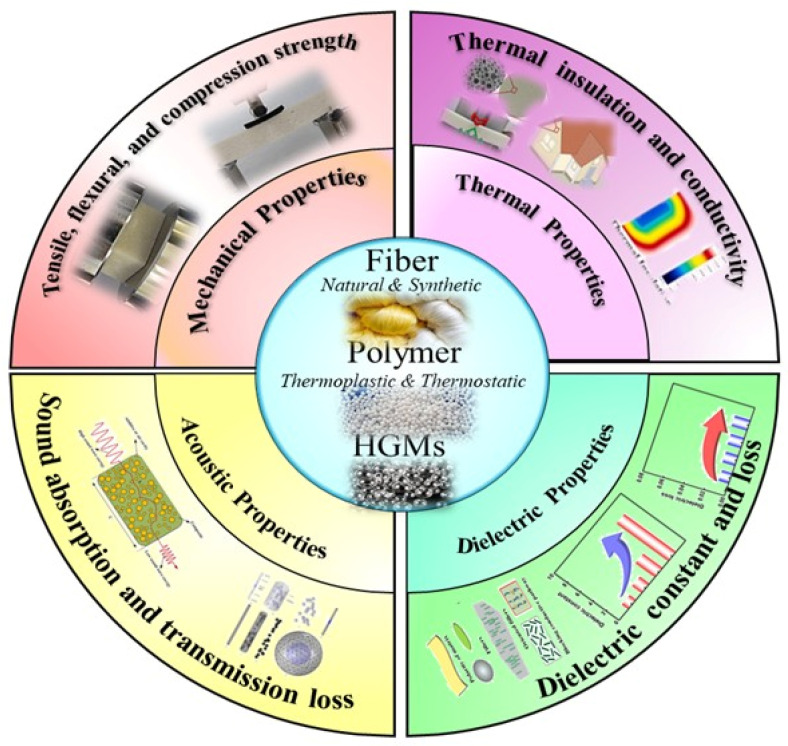
Basic focused areas and their categories are covered by this review.

Numerous researchers have studied the effect of HGMs on the FRPCs. This section discusses recent research works that have been performed using HGMs for different functional properties’ improvement of FRPC, basically mechanical, thermal, acoustic, and electrical properties, as shown in Figure 6, by integrating findings with scientific interpretation.

### 4.1. Mechanical Properties

The quantity of HGM filler plays a critical role in determining both the design (longitudinal and transverse) and physical properties of the composite, with a particularly significant impact on its mechanical performance [102,103,104]. HGMs also influence the compressive behavior of composites, often accelerating their deformation under load. Consequently, HGMs can be utilized to improve the compressive performance of lightweight composites compared to the resin matrix alone. A lot of the latest research and its findings on the effects of adding HGMs to the FRPC mechanical properties are presented in Table 1. The interaction between the resin and glass microspheres plays a crucial role in altering the composite’s overall properties [105]. Poor integration of HGMs into the matrix can cause them to behave like defects. At higher volume fractions, HGMs may agglomerate and create voids, resulting in stress concentrations that reduce the composite’s overall strength [106]. 

**Figure 7:**
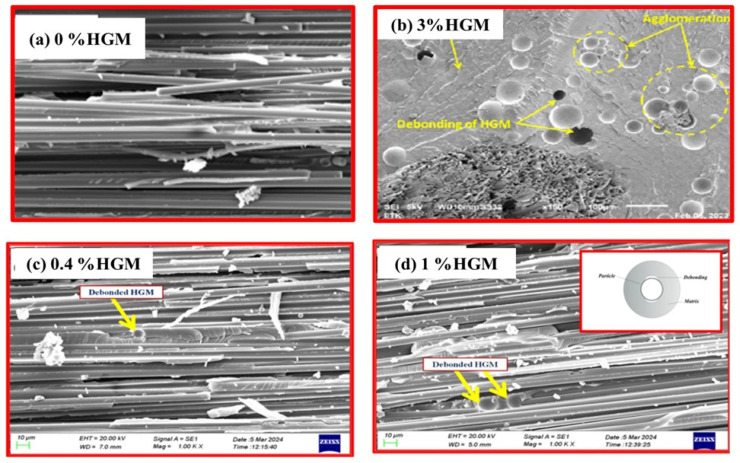
The SEM image of fiber, matrix and HGM interaction in composite at different concentrations of HGM. Reproduced from ref. [108] (**a**,**c**,**d**) [Copyright (2024), with permission from John Wiley]. Reproduced from ref. [117] (**b**) [Copyright (2023), with permission from John Wiley and Sons].

Optimal dispersion of HGMs leads to significant improvements in tensile strength and modules, demonstrating that incorporating HGMs at specific concentrations can enhance the composite’s mechanical properties. However, at higher concentrations, the tendency of HGMs to agglomerate generates stress concentration points that weaken tensile performance. This agglomeration disrupts the continuity of the matrix by restricting the mobility of molecular chains, resulting in inefficient load transfer and diminished mechanical behavior [114,115,116]. Scanning electron microscopy (SEM) images in Figure 7 show that the laminates confirm the uniform distribution of particles in specimens containing low concentrations of HGMs, whereas specimens with higher HGM concentrations exhibit noticeable agglomeration.

**Figure 8:**
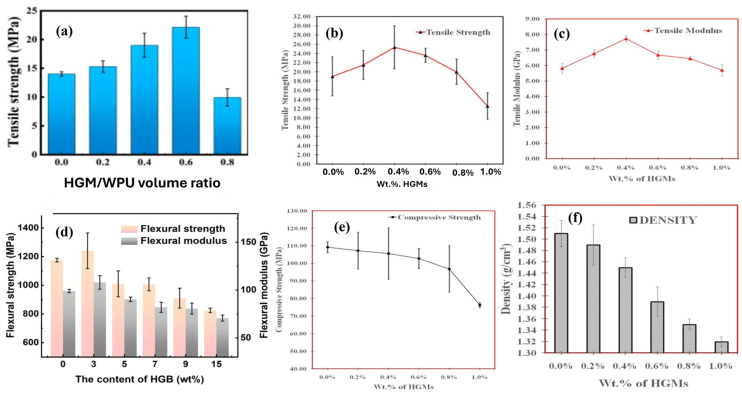
The effect of HGM content on FRPC mechanical properties. (**a**) Tensile strength in glass fiber [121]. Tensile strength, tensile modules, flexural strength, compressive strength and density in carbon fiber. Reproduced from ref. [108] (**b**,**c**,**e**,**f**) [Copyright (2024), with permission from John Wiley and Sons]. Flexural strength and modules. Reproduced from ref. [122]. (**d**) [Copyright (2023), with permission from John Wiley and Sons].

The intrinsic stiffness of HGMs is advantageous at lower concentrations but can become detrimental at higher loadings due to their susceptibility to buckling under compressive stress. In such cases, the particles tend to debond from the matrix rather than deform and absorb the load. Additionally, the incorporation of HGMs may reduce the crystallinity of the composite, hindering effective load transfer through the matrix and potentially resulting in lower tensile strength compared to the neat resin as shown in Figure 8. This underscores the importance of carefully controlling the HGM content to preserve structural integrity [118]. A positive effect of compatibilizer and surface modified HGMs was reflected on tensile strength as a result of improved interfacial adhesion with the matrix [109,119]. The particle size of HGMs also has a significant effect on the mechanical properties of the composite due to their different interfacial surfaces, wall thicknesses, and aspect ratios [120]. Additionally, Figure 8f illustrates the decrease in composite density with increasing weight percentages (wt%) of HGMs. This reduction is primarily attributed to the inherently low density of HGMs, which replace the denser matrix within the composite [108,109,111].

**Figure 9:**
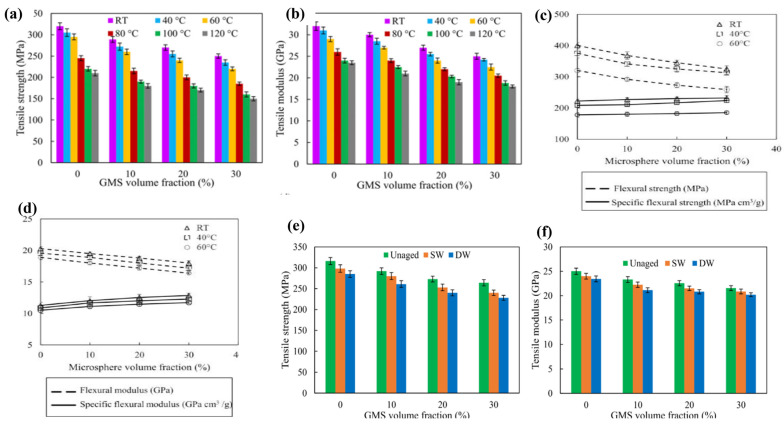
The effect of HGM content on different moisture and temperature conditions on the mechanical properties of FRPCs. Effect of temperature on tensile strength and modules. Reproduced from ref. [124]. (**a**,**b**) [Copyright (2024), The author(s), with permission from Paramasivam, A. et al., Journal of Composite Materials, 2024]. Flexural strength and modules. Reproduced from ref. [110]. (**c**,**d**) [Copyright (2024), The author(s), with permission from Paramasivam, A. et al., Journal of Materials Today: Proceedings, 2024]. Effect of moisture on tensile strength and modules. Reproduced from ref. [111]. (**e**,**f**) [Copyright (2024), The author(s), with permission from John Wiley and Sons].

Figure 9 below shows that prolonged exposure to moisture significantly deteriorates the interfaces between the HGM/matrix and fiber/matrix, resulting in reduced composite properties. Moreover, this interfacial degradation plays a key role in diminishing the composite’s overall stiffness [111,123]. The figure illustrates that the tensile strength and modulus of the composites significantly decrease at elevated temperatures compared to room temperature. These mechanical properties are predominantly governed by the fiber, which undergoes minimal degradation within this temperature range. In contrast, the degradation of flexural properties is more pronounced than the reduction in tensile strength, indicating that flexural performance is more strongly influenced by resin softening and the fiber/matrix interface, rather than by the fibers themselves [110,124].

### 4.2. Thermal Properties

Combining HGM as a filler and a matrix material reduces the weight of the composite material and improves the thermal insulation performance [125]. Numerous studies have investigated the effects of incorporating HGM fillers on the thermal conductivity and insulation properties of composites based on polypropylene, phenolic resin, epoxy resin, silicone rubber, and high-density polyethylene fiber-reinforced matrices [126,127,128,129,130]. Li and Pan highlighted trends in lightweight thermal insulators toward inorganic systems, composites, and microstructure optimization to improve fire resistance, strength, and reduce thermal conductivity [131]. Incorporating HGMs can thus enhance insulation while lowering composite weight.

Sun et al. investigated the enhancement of thermal insulation properties in composites composed of waterborne polyurethane (WPU) reinforced with glass fiber and HGMs as fillers. Their results showed that incorporating HGMs into WPU significantly improved the composite’s thermal insulation, reducing thermal conductivity by 45.2% at a volume ratio of 0.8 (HGM to WPU) compared to the composite without HGMs. However, increasing the HGM content beyond this ratio did not lead to further substantial decreases in thermal conductivity [121]. Once the HGM content reaches a threshold, the primary heat transfer pathways within the composite remain unchanged, resulting in no further improvement in the overall insulation performance [132].

**Figure 10:**
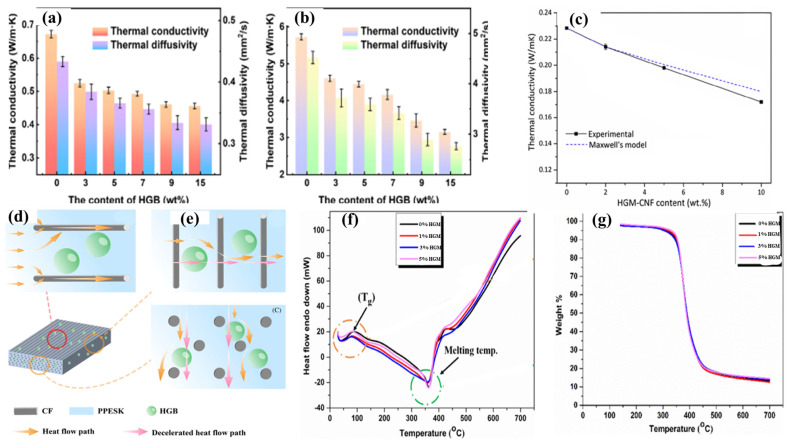
The effect of HGM on the thermal properties of FRPCs. Thermal conductivity and heat flow path in parallel and perpendicular direction of composite. Reproduced from ref. [122]. (**a**,**b**,**d**,**e**) [Copyright (2023), with permission from John Wiley and Sons]. (**c**) Thermal conductivity obtained from maxwell’s model and experimental work. Glass transition temperature, and TGA. Reproduced from ref. [117]. (**f**,**g**) [Copyright (2023), with permission from John Wiley and Sons].

Herrera-Ramirez et al. investigated the thermal properties of carbon nanofiber (CNF)/urethane acrylate resin composites incorporating HGMs. Using a chemical vapor deposition (CVD) method, CNFs were synthesized on the surface of HGMs before being added to the resin matrix. Their results showed that the thermal conductivity of the composites decreased by increasing HGMs content from 0% to 10%, including those with HGMs coated with CNFs. Specifically, the composite containing 10 wt% HGMs-CNFs (approximately 34 vol.% HGM) exhibited a thermal conductivity of 0.172 W/m·K, which is 25% lower than that of the neat resin [133]. Thermal conductivity is reduced due to the presence of contact between CNFs and between HGMs and the effect of the interfacial thermal resistance or Kapitza resistance between the CNFs and the matrix, the CNFs and the HGMs, and HGMs and the matrix [134,135]. Feng, J et al. demonstrated that the incorporation of HGMs significantly influences the thermal conductivity of carbon fiber/polyphthalazine ether sulfone ketone (CF/PPESK) composites. As the HGMs content increases, both the thermal conductivity and thermal diffusivity of the composite decline rapidly in directions parallel and perpendicular to the fiber orientation, as Figure 10 shows. At low HGM concentrations, the fillers are uniformly dispersed, preventing direct contact between particles and resulting in a linear decrease in thermal conductivity with increasing HGM content. However, beyond critical concentration, HGMs tend to agglomerate and form a network-like structure, hindering continuous heat flow through the composite. Additionally, poor compatibility between the inorganic fillers and resin matrix leads to increased defects and phonon scattering at interfaces, causing a rapid rise in interfacial thermal resistance and a consequent sharp decline in thermal conductivity [122].

Although the addition of HGMs reduces the thermal conductivity, as shown in Table 2, both the glass transition temperature (Tg) and melting temperature initially decrease, followed by an increase as the HGMs content continues to rise [117]. This behavior is attributed to increased HGM concentration, promoting particle agglomeration within the composite. These agglomerated particles act as heat sinks, slowing heat diffusion inside the matrix by absorbing heat and inhibiting crosslinking formation at the resin surface [118,136,137].

### 4.3. Acoustic Properties

While research on the combined use of fibers and HGMs within polymer matrices for acoustic applications remains limited, numerous studies have explored the incorporation of HGMs alone in composites for acoustic enhancement. Likewise, extensive investigations have examined the acoustic behavior of FRPCs, utilizing both natural and synthetic fibers for acoustic performance.

Dhilipkumar, T. et al. investigated the influence of banana fiber on the acoustic properties of epoxy matrix composites. Their findings indicated that fiber length, volume fraction, and composite thickness significantly affect both the sound absorption coefficient and sound transmission loss [144,145,146,147]. Short fibers have better sound absorption properties than the longest ones [148]. Acoustic absorption coefficients of the composites made of surface-treated natural fibers demonstrated superior sound absorption properties due to the high interaction with the matrix [149]. Synthetic fibers like glass and carbon fibers have also been used for the acoustic property evaluation of FRPCs [150,151].

**Figure 11:**
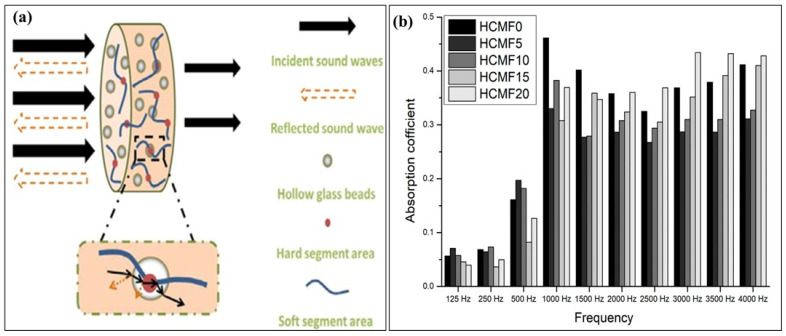
The role of HGMs in the sound insulation mechanism of composites. Reproduced from ref. [154]. (**a**) [Copyright (2020), with permission from John Wiley and Sons] and (**b**) Sound absorption graph of composite with HGM [156].

The incorporation of HGMs into composites significantly enhances sound insulation performance, primarily due to the combination of the matrix’s high damping capacity, the rigidity and hollow structure of the HGMs. As shown in Figure 11a, the HGMs either reflect or absorb the incident sound wave. When embedded in the resin, HGMs reflect high-frequency sound waves, while low-frequency waves transmit through the interface between the HGMs and the polyurethane matrix [152]. The incorporation of HGMs modifies the transmission angle of sound waves and extends their propagation path, thereby enhancing energy dissipation through the damping effect of the matrix.

It was observed that in Figure 11b, the epoxy material increased the sound transmission loss values with increasing reinforcement of HGM. The hollowness of the reinforcing material, its porous surface, and even incoherent interfaces found in certain zones of the composite also contributed to this condition. The gaps at the interfaces in certain zones of the composite were beneficial in preventing sound transmission [153]. Interactions between HGMs and the hard segments of the matrix reduce the material’s crystallinity and promote microphase separation, which contributes to increased sound attenuation. Furthermore, the greater rigidity of HGMs relative to the resin results in enhanced sound reflection, reducing sound transmission through the composite. The improved acoustic performance arises from the synergistic effects of microphase separation, interfacial interactions, and increased composite stiffness [154].

Yang, X. studied the effects of various parameters, including the densities of the composite matrix and HGMs, acoustic velocities in the polymer and inorganic particles, incident wave frequency, sound insulator thickness, and the diameter, volume ratio, and hollow ratio of HGMs on the sound transmission loss of composites using a finite element simulation model. Their findings indicated that the diameter, volume ratio, and hollow ratio of HGMs negatively affected sound insulation performance, whereas the thickness of the sound insulator was identified as the most influential parameter [155].

### 4.4. Dielectric Properties

Dielectric properties describe a material’s ability to store and transmit electric energy without conducting current. The dielectric constant, or relative permittivity, quantifies a material’s capacity to store electric charge when subjected to an external electric field, typically measured with the material placed between two parallel plates acting as a capacitor [157]. In recent years, FRPCs have been widely used as dielectric materials in electric and electronic parts in different areas of application [158]. Polymers possess desirable electrical properties, such as low dielectric loss, but have high dielectric constants, making them attractive for electronic components, energy storage, and sensing systems [159,160]. A large number of natural and synthetic fibers with distinct properties have been used to develop the dielectric materials, mostly using synthetic polymers as the matrix [161,162]. Since natural fibers are hydrophilic and have poor compatibility with synthetic polymers, chemical modifications are performed to obtain dielectric materials with desired properties [163].

The incorporation of fibers into polymer matrices generally improves the dielectric properties of FRPCs. The dielectric constant exhibits a positive correlation with temperature and a negative correlation with frequency. This behavior is attributed to increased charge carrier mobility and pronounced interfacial polarization within the FRPCs, especially at high temperatures and low frequencies [162,164]. Surface treatment of cellulosic fibers reduces their hydrophilicity by breaking hydrogen bonds and removing polar groups. This modification decreases orientation polarization within the fibers, leading to a lower dielectric constant in the treated fibers [158].

HGMs yield a hollow structure and contain air with a low dielectric constant, which can effectively reduce the density of the material and thus reduce the dielectric constant of the material [165,166]. The closed, compact shell of HGMs is primarily composed of SiO_2_, a material with low polarity. This characteristic contributes to reducing the dielectric constant of composites. Consequently, HGMs are well-suited as components in the fabrication of low-dielectric materials [167].

**Figure 12:**
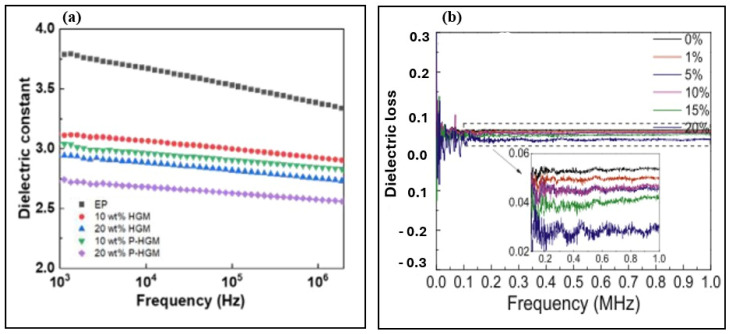
The role of HGMs in the dielectric constant. Reproduced from ref. [169]. (**a**) [Copyright (2022), with permission from John Wiley and Sons] and (**b**) in dielectric loss of composite [172].

Zhu et al. investigated the effect of HGMs with varying densities and volume fractions (0–60%) on the dielectric constant of epoxy matrix composites. They found that the dielectric constant of HGMs is lower than that of epoxy, resulting in a continuous decrease in the composite’s dielectric constant with increasing HGMs content or decreasing HGMs density, regardless of HGM type, as shown in Figure 12. Additionally, at the same filler content, composites filled with lower-density HGMs exhibited a more pronounced reduction in dielectric constant [168]. Similarly, Zhang et al. confirmed these findings by demonstrating that the dielectric constant of epoxy/HGMs composites decreased from 3.41 for pure epoxy resin to 2.77 at 20 wt% HGM loading, representing an 18.7% reduction. This decrease is primarily attributed to the increased air volume introduced by the HGMs, which possess large pore volumes and inherently low dielectric constants. Consequently, the overall dielectric constant of the composite is reduced [169].

The decrease in dielectric constant involves not only the incorporation of air but also the influence of interface polarization. The higher the frequency, the shorter the relaxation time. The polarization changes cannot be completed in such a short relaxation time, so the dielectric constant decreases [170]. Gao, G. et al. conducted similar research and found that the dielectric constant of composite materials remains relatively stable across increasing frequencies, demonstrating good frequency stability. However, dielectric loss exhibited noticeable fluctuations at low frequencies, attributed to the presence of air within the composites. Additionally, the dielectric loss gradually decreased with increasing HGMs content [171].

Ren, T. et al. [173] attempted to modify the surface of HGMs by introducing reactive vinyl groups to facilitate interfacial chemical reactions with the vinyl groups of a polybutadiene (PB) matrix.

This modification improved the organic–inorganic interface compatibility and increased interfacial peel strength. The chemical bonding between PB’s vinyl groups and the HGMs’ surface resulted in ultra-low dielectric loss. At lower filler loadings, surface-modified HGMs dispersed more uniformly within the matrix, reducing local defects and enhancing the composite’s dielectric properties. However, significant interfacial aggregation was observed at higher filler contents [173]. Similarly, Zhu B.L. et al. studied the effect of surface-modified HGMs in an LDPE matrix and found that the surface treatment improved the dielectric properties of the composites. This enhancement was attributed to improved interfacial adhesion between the filler and the polymer matrix [174].

## 5. Application Areas and Performance of HGMs-Filled FRPCs

The integration of HGMs into FRPCs has significantly expanded their applicability in high-performance and multifunctional materials across several industries. Their unique combination of lightweight structure, high compressive strength, low thermal conductivity, and excellent dielectric stability makes HGM-filled FRPCs highly attractive in sectors such as automotive, aerospace, marine, construction, and electronics.

In the automotive industry, HGMs have been incorporated into glass and carbon fiber-reinforced epoxy matrices to reduce component weight and improve fuel efficiency without compromising stiffness or strength. Jeong et al. reported a 15% improvement in thermal insulation and 20% density reduction in glass fiber–epoxy composites containing 10 wt% HGMs [175]. In aerospace applications, HGMs enhance stiffness-to-weight ratios and vibration damping, essential for fuselage panels and interior components; Tejasvi et al. observed up to 12% improvement in damping and 25% lower mass in HGM-modified carbon fiber laminates [108]. For marine and offshore structures, where weight and corrosion resistance are critical, HGM-reinforced FRPCs demonstrate reduced water absorption and enhanced hydrothermal stability. Anandakumar et al. found that HGM-modified glass fiber composites exhibited lower moisture uptake and improved retention of tensile strength after seawater immersion [111], while Wang et al. confirmed superior dimensional stability under cyclic wet–dry conditions [176].

In building and construction, FRPCs containing HGMs are used in acoustic and thermal insulation panels, sandwich structures, and façade systems. The hollow microstructure of HGMs acts as a sound and heat barrier, reducing heat transfer and airborne noise; Ajayi et al. reported that HGM/nano-clay cores in natural fiber composites improved sound absorption and reduced thermal conductivity by up to 18% [177]. Beyond structural use, HGMs impart desirable dielectric and electromagnetic (EM) shielding properties, which have attracted attention in electronic and telecommunication applications. Jiadong Lu et al. demonstrate that incorporating 10 wt% HGM into the epoxy resin system results in a dielectric constant of 2.39 at 10 MHz and a dielectric loss of 0.018, indicating improved insulation performance for high-density interconnect and substrate-like printed circuit board applications [172]. The combination of low dielectric constant, low loss tangent, and high thermal stability makes HGM-modified FRPCs attractive for applications where signal integrity, heat dissipation, and EM protection are critical.

## 6. Challenge and Future Prospects

The integration of FRPCs faces several well-documented challenges. Previous studies have highlighted issues such as agglomeration due to poor dispersion, weak interfacial bonding with the polymer matrix, and the inherent fragility of microsphere walls, which can fracture under processing shear, compromising both weight reduction and mechanical performance. Researchers have proposed strategies including surface functionalization, coupling agent treatments, nano-coatings, hybrid filler systems, and optimized processing techniques to enhance dispersion, interfacial adhesion, and mechanical integrity.

Building on these insights, we propose that future research should focus on the design and development of engineered HGMs with tailored surfaces, such as dual-functional coatings that combine nano-roughened inorganic layers for mechanical interlocking with functional polymer layers for chemical bonding, fiber-specific surface chemistries tailored to the type of reinforcing fiber, stimuli-responsive coatings that adapt during processing to reduce agglomeration and lock in place after curing, hierarchical micro/nano-texturing to enhance interfacial interlocking and shear strength without increasing weight, and bio-compatible functionalization for biopolymer matrices to enable lightweight, eco-friendly, multifunctional composites.

Such engineered microspheres, when incorporated into FRPCs, have the potential to extend their utility far beyond structural reinforcement. Combining HGMs with nanofibers and biopolymers could lead to composites suitable for advanced applications, including thermal management, smart biomedical devices, and multifunctional lightweight materials. This multi-disciplinary approach bridges structural performance and functional adaptability, paving the way for next-generation FRPCs that are both mechanically robust and functionally versatile.

## 7. Conclusions

HGMs have emerged as highly versatile functional fillers for FRPCs, offering a unique combination of low density, high specific strength, thermal insulation, and tailored dielectric behavior. Their spherical geometry and tunable physical and chemical properties enable significant improvements in mechanical performance, including tensile, flexural, and compressive strength when optimally dispersed within the polymer matrix. Beyond mechanical reinforcement, HGMs contribute to enhanced thermal stability, reduced thermal conductivity, superior acoustic damping, and desirable dielectric characteristics, making them suitable for applications across automotive, aerospace, marine, construction, and electronics industries.

The selection of both polymer matrix and HGM type should align closely with the targeted application, ensuring optimal performance. Notably, glass beads used as HGMs can be produced from waste materials or as byproducts of critical industrial processes, offering high recyclability and minimal environmental impact. This sustainability aspect positions HGM composites as eco-friendly, green materials with the potential to replace metals or conventional composites in various sectors. However, the performance benefits of HGMs depend heavily on factors such as microsphere wall thickness, particle size distribution, surface treatments, filler loading, fiber–matrix interfacial adhesion, and processing conditions. Achieving the right balance between weight reduction and mechanical integrity is crucial, as excessive HGMs content may lead to matrix embrittlement or decreased impact resistance. Recent advancements in manufacturing techniques, including surface functionalization and hybrid filler systems, present new opportunities to engineer FRPCs with multifunctional capabilities. Nonetheless, challenges remain in ensuring uniform dispersion, preventing microsphere breakage during processing, and optimizing compatibility across diverse fiber–matrix systems.

## Figures and Tables

**Table 1:** Mechanical properties of FRPCs using HGM as a filler.

Types of Fiber and Polymer Matrix	HGMs Volume Fraction	Maximum Value of				Reference
		Tensile Strength	Tensile Modulus	Flexural Strength	Compression Strength	
Jute, hemp, flax/Polypropylene	0.0–3.0%	40.7 MPa flax at 1.5% HGM	8.6% flax at 1.5% HGM	19% Jute at 1.5% HGM	-	[107]
Carbon fiber/epoxy resin	0.0–1.0%	25.4 MPa at 0.4%	7.4% at 0.4%	-	High at 0% HGMs	[108]
Carbon fiber/polypropylene	0.0–40%	85.03% MPa at 10%HGMs with 8% fiber	6.72%, at 40% HGMs with 8% fiber	87.2MPa at 10% HGMs with 8% fiber	-	[109]
Glass fiber/epoxy under high temperature (RT-60 °C)	0.0–30%	Declined by 8% from RT	Declined by 6% from RT	Decrease of 24% from RT		[110]
Glass fiber/epoxy under moisture (sea water SW and distilled water DW)	0.0–30%	Reduction 13% DW and 9% SW	-	Reduction 24% DW and 11% SW	-	[111]
piassava fiber/polypropylene	0.0–5%	33.29 MPa at 0%	2055.3 MPa at 5%		59.22 MPa at 0%	[112]
Bamboo fiber/polypropylene	10 wt%	Enhanced by 14.38% from base matrix at 20 wt% fiber	Enhanced by 65.55% from base matrix at 20 wt% fiber	Increases by 18.4% from base matrix at 20 wt% fiber	Increases by 39.2% from base matrix at 20 wt% fiber	[113]

**Table 2:** The effect of HGMs on thermal and acoustic properties of FRPCs.

Types of Fiber and Polymer Matrix	HGM Volume Fraction	Acoustic Properties	Thermal Properties		References
			Thermal Conductivity	Thermal Insulation	
PDMS (Polydimethylsiloxane) + HGMs	0–17 wt%	Decrease from −29 dB (pure PDMS) to −26 dB (at 17 wt% HGMs)	Reduced by 31% (from ~0.2 W/mK)	Significantly improved	[138]
Glass fiber fabric/Waterborne PU	0–0.8 HGMs/WPU (volume ratio)	Not studied	A 45.2% reduction was observed, decreasing from 0.21 at 0% HGM to 0.1154 W/(m·K) at a 0.8 HGMs ratio	A 17.74 °C temperature difference (25.3% insulation) was achieved at 70 °C (ratio 0.8)	[121]
Cotton Fabric/Acrylic Binder Composite	20 wt%	Excellent absorption noise reduction coefficient (0.303) across frequency (0–3500 Hz).	Attributed to a considerably low value because of the hollow structure	Remarkable 78% enhancement in thermal resistance; acts as an effective heat barrier.	[139]
Surface-modified HGMs (KH560)/Epoxy Resin	67 vol%	Exceptionally low permittivity (Dk = 2.3) at 110 MHz	Reduced to 0.14 W·m^−1^·K^−1^ (from 0.24 for neat epoxy) due to hollow, gas-filled microspheres acting as thermal barriers	Excellent (42% reduction) in conductivity compared to neat epoxy, making it suitable for electronic packaging.	[140]
Alkali-treated Jute/Epoxy	2.5 vol.%	-	Decreased (Low thermal conductivity confirmed)	Improved (Stable up to 540.0 K/266.9 °C)	[141]
Glass fiber fabric/Epoxy	7% by weight	Max absorption coefficient of 0.18 at 6300 Hz, showing no improvement over the reference value of 0.1	Slightly higher than the reference value, 0.484 ± 0.005 W/mK compared to 0.480 W/mK	No improvement, as agglomeration limited HGMs’ contribution to thermal insulation	[142]
Phenolic Resin/HGMS	20 wt%	-	Reduced at 0.129 W·m^−1^·K^−1^, lower than neat resin due to porosity; higher loadings increased from microsphere crushing	Excellent (~46.7% lower than neat resin)	[143]
HDPE/Hollow Glass Microspheres (HGMs)	1 wt%, 5 wt%	Increased absorption coefficient, best for Internal Mixer (IM1) at 1% HGMs (0.85 at 1250 Hz).	Reduction in thermal conductivity for similar systems	Improved thermal stability with higher decomposition temp than neat HDPE; best at 5% HGMs.	[136]

## Data Availability

No new data were created or analyzed in this study. Data sharing is not applicable to this article.
